# Improved the biocompatibility of cancellous bone with compound physicochemical decellularization process

**DOI:** 10.1093/rb/rbaa024

**Published:** 2020-08-30

**Authors:** You Ling, Weikang Xu, Lifeng Yang, Changyan Liang, Bin Xu

**Affiliations:** r1 National Engineering Research Center for Human Tissue Restoration and Function Reconstruction, School of Materials Science and Engineering, South China University of Technology, Guangzhou Higher Education Mega Centre, Panyu District, Guangzhou, Guangdong 510006, China; r2 Department of Scientific Research, National Engineering Research Center for Healthcare Devices, Guangdong Key Lab of Medical Electronic Instruments and Polymer Material Products, Guangdong Institute of Medical Instruments, Guangdong Academy of Sciences, No. 1307 Guangzhou Avenue Central, Tianhe District, Guangzhou, Guangdong 510500, China; r3 National Engineering Laboratory for Regenerative Implantable Medical Devices, R&D Center, Grandhope Biotech Co., Ltd, Guangzhou, Guangdong 510530, China; r4 Department of Biosecurity Evaluation, Guangdong Medical Devices Quality Surveillance and Test Institute, No. 1 Guangpu West Road, Huangpu District, Guangzhou, Guangdong 510663, China; r5 Department of Gynecology, Third Affiliated Hospital of Sun Yat-sen University, No.600 Tianhe Road, Tianhe District, Guangzhou 510630, China

**Keywords:** extracellular matrix, cancellous bone, physicochemical decellularization, biocompatibility

## Abstract

Due to the unique microstructures and components of extracellular matrix (ECM), decellularized scaffolds had been used widely in clinical. The reaction of the host toward decellularized scaffolds depends on their biocompatibility, which should be satisfied before applied in clinical. The aim of this study is to develop a decellularized xenograft material with good biocompatibility for further bone repair, in an effective and gentle method. The existing chemical and physical decellularization techniques including ethylene diamine tetraacetic acid (EDTA), sodium dodecyl sulfate (SDS) and supercritical carbon dioxide (SC-CO_2_) were combined and modified to decellularize bovine cancellous bone (CB). After decellularization, almost 100% of ɑ-Gal epitopes were removed, the combination of collagen, calcium and phosphate was reserved. The direct and indirect contact with macrophages was used to evaluate the cytotoxicity and immunological response of the materials. Mesenchymal stem cells (MSCs) were used in the *in vitro* cells’ proliferation assay. The decellularized CB was proved has no cytotoxicity (grade 1) and no immunological response (NO, IL-2, IL-6 and TNF-α secretion inhibited), and could support MSCs proliferated continuedly. These results were similar to that of commercial decellularized human bone. This study suggests the potential of using this kind of combine decellularization process to fabricate heterogeneous ECM scaffolds for clinical application.

## Introduction

Extracellular matrix (ECM) scaffolds are the direct or end result of a decellularization process of tissues or organs, which have been used in more than 8 million patients to date [1]. Critical size bone tissue defects cannot self-repair completely, and causing a huge burden on economy and society [2]. Safe and effective bone replacement procedures are very important, so that autograft, allograft and artificial materials have been applied. However, limited source of autograft, and immune rejection of allograft limit the application of their bone repair [3]. With the development of regenerative medicine strategies, various artificial biodegradable materials have been used to simulate natural bone, including collagen, alginate, poly(lactic-*co*-glycolic acid) and polylactic acid [4–6]. In fact, there is still no tissue engineering method that can design bionic whole human-sized bone structure and construct the unique three-dimensional microenvironment that contains ECM properties completely that are essential for bone repair. ECM is known to have 3D ultrastructure, complex composition and regulatory functions, which is more suitable than artificial biodegradable materials [7].

The feasibility of decellularization of some tissues including Achilles tendon or rotator cuff have been verified [8, 9]. During the decellularization process, physical methods such as freeze–thaw cycles and supercritical fluids, chemical and biologic methods such as triton X-100 (non-ionic detergents), ethylene diamine tetraacetic acid (EDTA, chelating agents), latrunculin B (marine toxin) and DNase I (enzymatic agents) are used in combination or alone to lyse cells and remove cell debris [10–13]. For example, biomaterials could be compatible with supercritical carbon dioxide (SC-CO_2_), which leaves no toxic residues. Under moderate conditions (32°C, 7.4 MPa), the cells in aortic tissue could be removed by SC-CO_2_ effectively in 15 min [14]. The tissue engineering scaffolds should be non-toxic and non-immunogenic basically, which provide suitable microenvironment to guide cells adhesion, growth, proliferation and differentiation. However, different decellularization process could affect the cytotoxicity of xenogeneic material. For example, some porcine decellularized scaffolds, meshes and powders used in patients [15]. Cytotoxicity could due to the xenogenic bone them, or biochemical reagents retained during decellularization process [16]. The implantation of scaffolds could cause immune response partially mediated by macrophages [17]. In more detail, it may be caused by incomplete decellularization caused by the retention of cells or galactose-*α*-(1,3)-galactose (Gal epitope) on the scaffolds [18]. Molecules, cytokines, cryptic peptides and so on released from decellularized scaffolds could also cause immune response. It is necessary to deal with process which is required for complete decellularization and causes minor changes in tissue properties.

Previous studies have shown that proper physical and chemical treatments can effectively improve the biocompatibility of ECM [19–22]. We postulate that good biocompatible decellularized bovine cancellous bone (CB) grafts could be developed by a combination of commonly used decellularization methodologies including SC-CO_2_. To determine the biocompatibility of decellularized bone, it is necessary to evaluate the effect of decellularized scaffolds and materials release from scaffolds on cellular toxicity and immunity *in vitro*.

Macrophages are often used to study the immune-specific response, which participate in the host’s response to the implanted scaffolds. Substances that are harmless to cells *in vitro* may be inert *in vivo*, even in long-term experiments [23]. In order to further judge whether the decellularized scaffolds have the potential for clinical application, it was verified by cells proliferation test *in vitro*. Based on the results of cell experiments, we compared the decellularized scaffolds with two kinds of bone graft products. These results implicate that the decellularized scaffolds prepared by this method have the potential for clinical bone repair.

## Material and methods

### Decellularization of CB

**Figure 1:**
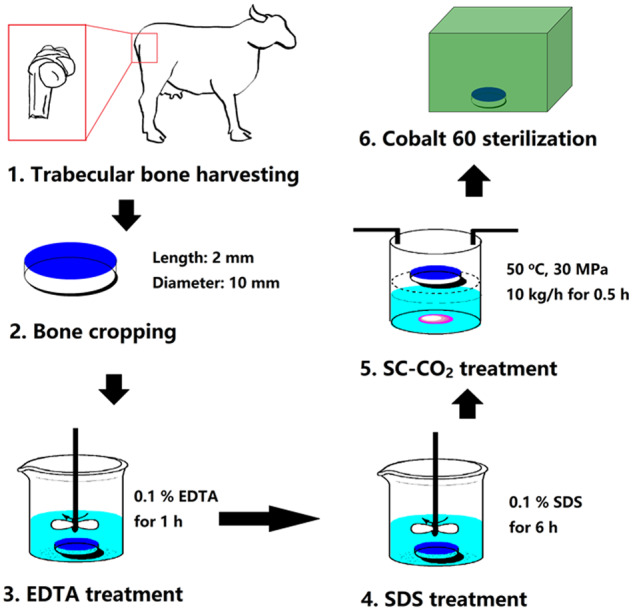
Schematic diagram of the fabrication process of decellularized bovine bone scaffolds.

Decellularized cancellous bone (DCB) were prepared using an improved method previously reported [14, 24]. Briefly, the trabecular bone in the subchondral region of the arm of cows aged from 2 weeks to 4 months were taken. The blocks were made into cylinders with a length of 2 mm and a diameter of 10 mm and were washed with high-speed flow water. Then scaffolds were detergent decellularized [0.1% EDTA (wt/vol) in PBS] for 1 h and detergent [10 mM Tris, 0.1% sodium dodecyl sulfate (SDS, wt/vol)] for 6 h at room temperature, washed with PBS several times until no bubbles were seen. SC-CO_2_ treatment was performed with minimum pressure of 8 MPa and maximum pressure of 15 MPa at 37°C (Speed SFE 4, Applied Separations, USA) system. The scaffolds were exposed to a pressure of 15 MPa for 10 min with flow rate of 10 kg/h. At the end of the SC-CO_2_ treatment, the vessel was slowly depressurized over 5 min. Pressurization and depressurization time was not added to the processing time. To make sure the implants are sterile, scaffolds were sterilized by cobalt-60 and rinsed in PBS, and incubated in culture medium overnight before cells experiment (Fig. 1).

### SEM analysis

The scaffolds were imaged by SEM to characterize the surface morphology and pore structure. Imaging by sputtering gold plating, using a lens detector with an acceleration voltage of 5 kV at a calibrated magnification (Tescan Mira XMU, Tescan USA Inc.).

### FTIR and Raman analysis

For FTIR analysis, the samples were mixed with KBr and subjected to a pressure to produce KBr pellets. On the Nicolet Avatar 330 FTIR spectrometer equipped with air-cooled deuterated triglycine sulfate, the FTIR spectrum in the 4000–400 cm^−1^ region was recorded at the temperature of 25 ± 10°C. These replicates were averaged and then used. The spectrum was analysed by ORIGIN 6.0 software (OriginLab Corporation, MA, USA).

The RM2000 micro-laser Raman spectrometer of Renishaw Public Co., Ltd. was used for Raman analysis. Laser source wavelength: 514.5 nm, total power: 20 MW, sample power: 5.8 nm, scanning frequency: 2 times/10 s, test diameter: 2 μm.

### XRD analysis

For XRD analysis, the samples are mixed, freeze-dried, milled into fine powder, then agate mortar is used and stored in a dryer until use. XRD analysis was carried out by using X-ray Diffractometer (X’ PERT PRO-PANACTIAL, PHILIPS) and Cu Kα radiation with a step of 0.05^°^ and a time/step of 10.16 s.

### *α*-1,3 Gal ELISA test

CB and DCB were homogenized after rinsing and weighing in cold PBS (0.02 M, pH 7.0–7.2). Cut the tissue into small pieces and homogenate in PBS on ice. The suspension was treated by ultrasonication and centrifuged for 1500 g for 15 min, and the supernatant was taken for analysis. The ELISA test for *α*-1,3 Gal was carried out using a commercially available kit (Alpha-Galactosyl ELISA Kit, BlueGene Biotech, Shanghai, China) as previously reported [25]. The supernatant of 100 μl from four tissues (*n* = 8 per tissue) was loaded into the microtiter-plate wells which was pre-coated with antibody. The positive control group of pig liver was labeled as PC, and the negative control group of human placenta was labeled as NC. The conjugate (50 μl/well) was added and incubated in the dark at 37°C for 1 h. Wash the micro-titer plate, turn it upside down and suck it dry. 15 min was incubated with 100 μl/well horseradish peroxidase substrate buffer at room temperature and dark.

The terminating fluid was added (50 μl/well), and the Optical Density (OD) at 450 nm was read by a micro-titer plate reader (Epoch R, BioTek) immediately.

### Cytotoxicity assay

Mouse peritoneal macrophages (PM) were purchased from American Type Culture Collection (ATCC, Manassas, VA, USA). Four categories of materials were used to study the cells response. Except the CB and DCB group, the commercial decellularized human bone (Cancellous bone strip, Beijing Xinkangchen Medical Science and Technology Development Co., Ltd., China, named MC1) and commercial biological ceramic artificial bone (DONGBO, Hunan Gongchuang Biofunctional Materials Co., Ltd, China, named MC2) were used as two control group.

To evaluate PM responses to different scaffolds at direct contact, all scaffolds (named D-CB, D-DCB, D-MC1 and D-MC2, respectively) balance 15 min in 24-well ultra-low attachment plates. PM (1 × 10^6^ cells) was inoculated on the scaffolds and allowed to attach under 37°C and 5% CO_2_ for 1 h, and the culture medium was adjusted to a final volume of 1 ml, as described previously [26].

In order to evaluate the response of PM to different scaffolds during indirect contact, all scaffolds (named I-CB, I-DCB, I-MC1 and I-MC2) were placed in the apical chamber (micropore) of 24-well ultra-low attachment plate (Millipore) and separated from PM in 250 μl medium. PM (1 × 10^6^ cells) was inoculated in the basolateral chamber and the culture medium was 750 μl. The scaffolds and cells were co-cultured in 37°C and 5% CO_2_ for 2 days, and the culture medium was changed on the first day.

PM (1 × 10^6^ cells) was seeded in basolateral chamber in 750 μl of culture medium. M0 controls were obtained by treating PM with culture medium alone. M1 (pro-inflammatory macrophage) controls were obtained by treating cells with 20 ng/ml of γ-IFN and 100 ng/ml of LPS.

After 48 h of incubation, the fresh culture medium 100 μl/well Containing Cell counting Kit 8 (CCK-8, Dojindo, Japan) was added and incubated at 37°C for 1 h. The absorbance of the supernatant was measured by microplate instrument at 450 nm. The formula of relative growth rate (RGR) is: RGR (%) = (OD value of experimental group/OD value of negative control group) × 100. Evaluation of cytotoxicity of samples according to the standards of American Pharmacopoeia [27]. Briefly, (i) RGR ≥ 75%, cytotoxicity grade 0 or 1, qualified; (ii) 74% ≥ RGR ≥ 50%, cytotoxicity grade 2; (iii) RGR ≤ 49%, cytotoxicity grade 3–5, unqualified.

### Cytokines and NO secretion

After 48 h incubation, the supernatants were collected. The corresponding detection kit was used to quantify cytokines, and 3 repeats were set for each test. Mouse interleukin-2 (IL-2), interleukin-6 (IL-6), interleukin-17A (IL-17A), interferon-gamma (IFN-γ), tumor necrosis factor-α (TNF-α) and interleukin-10 (IL-10) ELISA kits were purchased from eBioscience. The concentration of cytokines and the number of cells in each group were normalized.

NO assay was carried out according to the report described previously [28]. The optical density at 550 nm was measured by mixing an equal amount of medium (50 μl) with Griess reagent (1% sulfonamide and 0.1% naphthalene ethylenediamine in 5% phosphoric acid). Total RNA was extracted from macrophages by acid guanidine isothiocyanate-phenol method. Macrophages were dissolved with Isogen (Wako Purechmemical Co., Ltd).

### *In vitro* cells proliferation on scaffolds

Mouse mesenchymal stem cells (MSCs) were purchased from ATCC. The scaffolds (CB, DCB, MC1 and MC2) were placed in a 24-well TCPS plates, and the bottom of the scaffolds was pre-coated with aseptic agarose gel. 50 μl cell suspension (4 × 10^6^ cells/ml) was inoculated on the scaffold and cultured in DMEM at 37°C, 5% CO_2_ and 10% fetal bovine serum. After 3 days, the cells culture medium (1 ml) was changed to remove the unadherent cells, and then the medium was changed every 3 days. According to the manufacturer’s instructions, the cell number was determined by DNA analysis using Sigma quantitative kit (Sigma-Aldrich, Singapore). By measuring the number of DNA, the exact number of committed cells can be calculated using the DNA conversion rate of each cell’s 6.6 pg. The morphology of the cell-scaffold composites (CB, DCB and MC1) for day 7 was characterized by SEM.

### Statistical analysis

There are five samples in each group (*n* = 5), and the data of each group is shown in the figure. Statistical analysis was performed using ANOVA with LSD or Bonferroni’s post-hoc test and a two-tailed Student’s *t*-test for paired samples analysis (SPSS 21.0). It is considered that there is a significant difference between conditions if *P* < 0.05 or *P* < 0.001.

## Results

### Physicochemical properties of scaffolds

**Figure 2:**
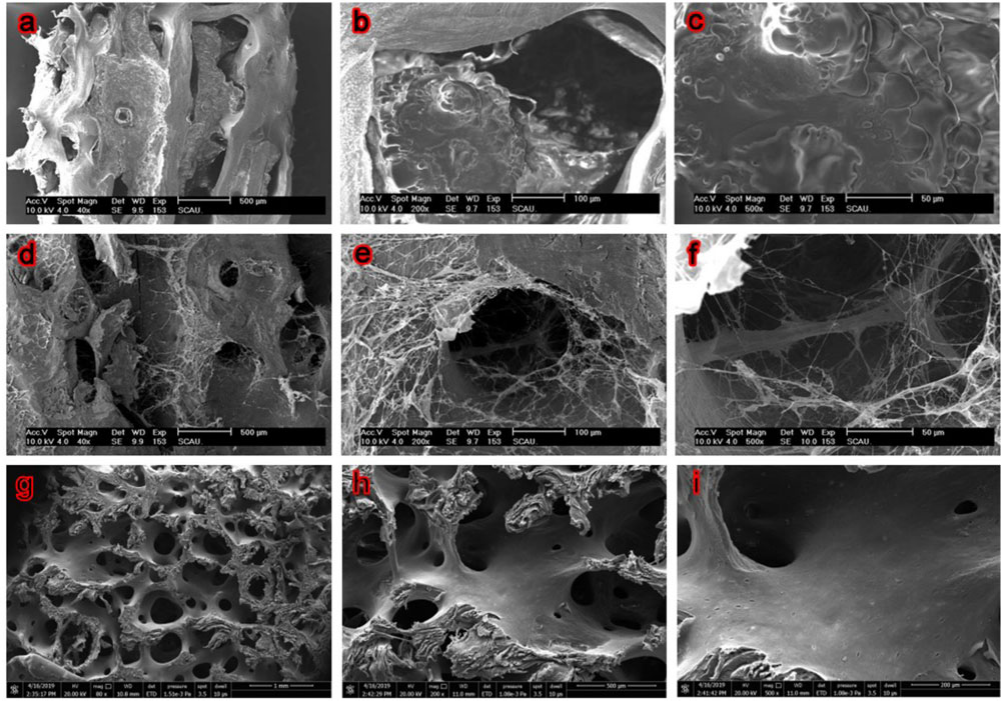
SEM Images show the microstructures of fresh CB, DCB and MC1. **a**–**c** Fresh CB, **d**–**f** DCB, **g**–**i** MC1, ×40, 200 and 500 magnification. After decellularization, the network of DCB was clearer, which is similar to the MC1.

SEM revealed remarkable differences in the microstructures of CB, DCB and MC1 surfaces (Fig. 2). The CB shows dense surface, with a large number of solid materials filling in micropore which results in poor porosity. In the enlarged micropores images, a large number of cell community-like substances and impurity could be found on the surface and in the pore structure of bone matrix (Fig. 2b and c), and the porosity was difficult to characterize. In contrast, the network of DCB was clearer, which has many micropores, and has better porosity (76.17%, characterized by X-ray 3D scanning). Only limited fibrous substances were found and no obvious residual substances were found in the micropores. Compared with DCB, the network of MC1 is also clear and the micropores are smaller.

**Figure 3:**
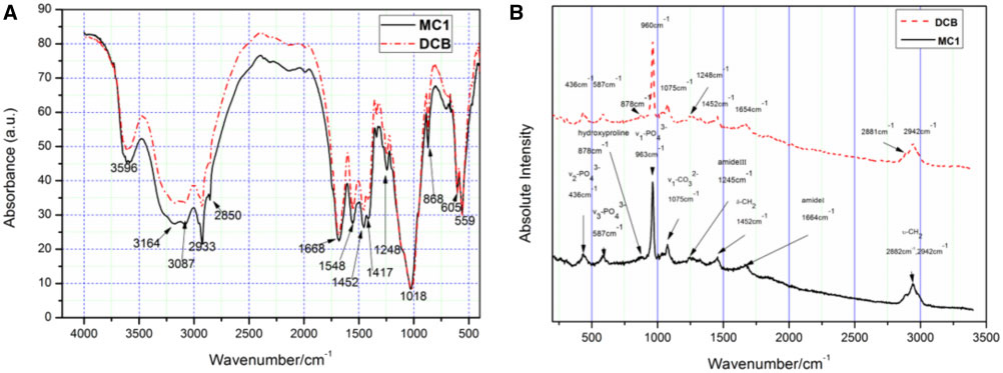
FTIR (**a**) and Raman (**b**) spectra show that the chemical composition of DCB was similar to MC1.

Fig. 3a shows the FTIR spectra of DCB and MC1. The broad absorption band at 3000–3500 cm^−1^ corresponds to the stretching vibrations of the OH– group from the H_2_O molecule and the N–H bond from the amide [29]. The peaks of 2933, 2850 and 1452 cm^−1^ can be classified as CH_2_ vibration, mainly from lipids, followed by proteins, carbohydrates and nucleic acids. The peak at 1417 cm^−1^ is caused by the vibration of CH_3_ and carbonate m3 [30]. In the range of 1200–2000 cm^−1^ can be specified to the absorbed from the organic matrix of bone. The peak at 1668 cm^−1^ is related to the C=O of amide I, and the peak at 1548 cm^−1^ is related to the N–H and C–N groups of amide II. The amide III (including C–N, N–H and C–C vibrations) brings the contribution spectrum by having a wide absorption at 1248 cm^−1^. The absorption observed in the range 1200–400 cm^−1^ is related to the lattice vibration of bone mineral composition. The strong peak at 1018 cm^−1^ can be attributed to the v1 and v3 vibration modes of ${\text{PO}}_{4}^{3-}$. The sharp peak at 868 cm^−1^ is a characteristic of absorbing v2 ${\text{CO}}_{3}^{2-}$ mode.

Fig. 3b shows the Raman spectra of DCB and MC1. Include v1 ${\text{PO}}_{4}^{3-}$ vibration mode at 960 cm^−1^ in hydroxyapatite molecule and v3 ${\text{PO}}_{4}^{3-}$ at 1041 cm^−1^, v1 ${\text{CO}}_{3}^{2-}$ at 1075 cm^−1^ (where ${\text{PO}}_{4}^{3-}$ replaces ${\text{CO}}_{3}^{2-}$ with B type) [31]. 878 cm^−1^ corresponds to the weaker band of C–C stretching vibration in proline and hydroxyproline, and 1004 cm^−1^ corresponds to C–C stretching vibration in phenylalanine, which represents the organic component of bone matrix [31]. Band at 1248 cm^−1^ correspond to amide III, and band at 1452 cm^−1^ correspond to of δ(CH_2_) and δ(CH_3_) modes. The spectral region of 1600–1700 cm^−1^ is the definition for evaluating the structural integrity of collagen fibers. This region corresponds to 1654 cm^−1^ of amide I [32]. Bands at 2881 and 2942 cm^−1^ can be attributed to the stretching vibrations of CH_3_ and CH_2_ in collagen [33].

**Figure 4:**
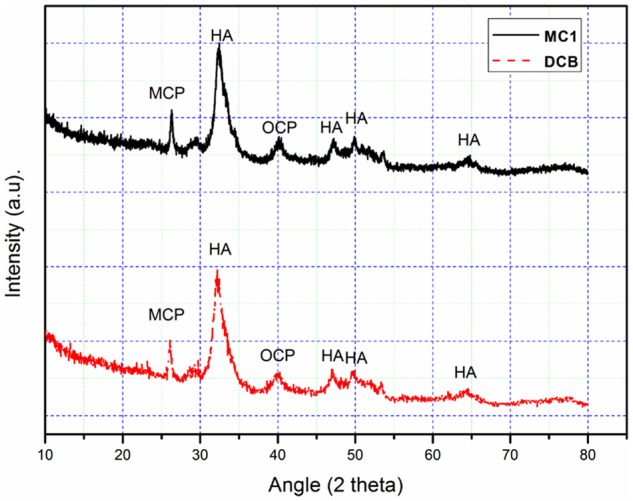
X-ray diffractogram show the crystal structure of DCB was similar to MC1.

The XRD spectrum of DCB and MC1 all shows the contours of (113), (131), (321), (134), (006) and (244) reflection rays, and their corresponding peaks are 26.12, 32.17, 40.08, 47.03, 49.78 and 64.39 (Fig. 4). There are several diffraction peaks, which clearly belong to the characteristic peaks of calcium phosphate, calcium carbonate, octacalcium phosphate and calcium magnesium phosphate, respectively [34], which reveal that the crystalline structure of DCB and MC1 was poor.

To further investigate the effectiveness of decellularized process, the α-Gal content of scaffolds was also detected. The α-Gal content in CB and PC was 0.076 × 10^14^/mg and 1.429 × 10^14^/mg, respectively. However, the α-Gal content in DCB and NC were not detected. Under this decellularization condition, the treatment of CB tissues can effectively remove α-Gal xenoantigen (Table 1).

**Table 1:** Quantification of α-1,3 gal content (*P* < 0.001)

Sample	Average content of gal-BSA (µg/mg)	Average number of gal antigen (*10^14^/mg)	SD (*10^14^/mg)	CV%
CB	0.076	0.138	0.008	5.84
DCB	Not detected	Not detected		
PC	1.429	2.600	0.989	38.023
NC	Not detected	Not detected		

### Cytotoxicity of scaffolds

*In vitro* toxicity studies of scaffolds were conducted using the CCK-8 assay with PM. To evaluate the temporal effects of scaffolds on macrophage’s cytotoxicity, the PM were seeded on scaffolds. At the time points, OD values of native D-CB was significantly lower than M0 and M1 (*P* < 0.05). The cytotoxicities were grade 1 for D-DCB, D-MC1 and D-MC2, and grade 3 for the native D-CB (Table 2).

**Table 2:** Relative growth rates (RGR) and cytotoxicity grades (CTG) of PM direct and separate cultured with scaffolds after 48 h

Group	OD value	RGR (%)	CTG
M0	1.813 ± 0.004		
M1	1.549 ± 0.075	85.40	1
D-CB	0.846 ± 0.003	46.63	3
D-DCB	1.460 ± 0.055	80.51	1
D-MC1	1.480 ± 0.287	81.62	1
D-MC2	1.572 ± 0.098	86.69	1
I-CB	0.922 ± 0.032	50.85	2
I-DCB	1.812 ± 0.027	99.91	1
I-MC1	1.589 ± 0.124	87.60	1
I-MC2	1.594 ± 0.027	91.05	1

After the decellularization process, some residual chemicals may be retained in bone material. To investigate the cytotoxicity of substances released from bone material, PM was separate cultured with scaffolds. At the time points, OD values of native I-CB was also lower than the others significantly (*P* < 0.05). The cytotoxicities were grade 1 for I-DCB, I-MC1 and I-MC2, and grades 2 for the I-CB (Table 2). Combined results of temporal changes in cytotoxicity indicate that DCB was not cytotoxic.

### Cytokines and NO secretion of MP

**Figure 5:**
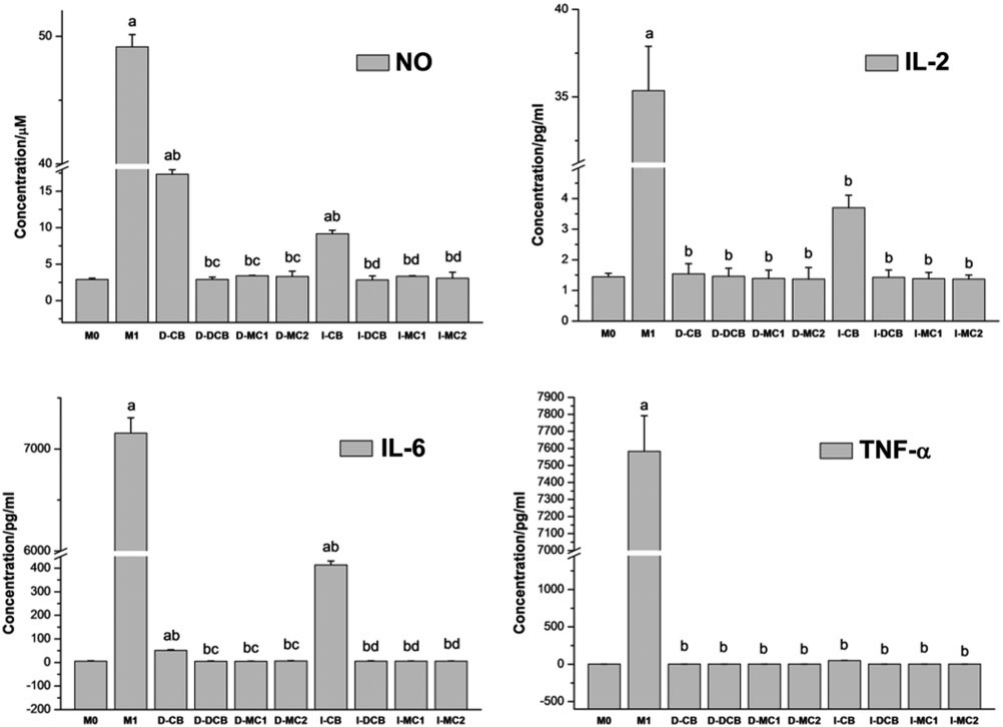
The NO and Cytokine profiles (IL-2, IL-6, IL-17A, IL-10, TNF-α and IFN-γ) of PM direct and separate cultured with scaffolds for 48 h, which show that CB induced NO, IL-2, IL-6 and TNF-α production significantly, but DCB inhibited the secretion of these three cytokines and NO significantly (^a^*P* < 0.05 vs M0; ^b^*P* < 0.05 vs M1; ^c^*P* < 0.05 vs D-CB; ^d^*P* < 0.05 vs I-CB).

One of the main functions of macrophages is to secrete a wide range of cytokines to cope with environmental changes, which coordinate tissue regeneration with other types of cells. Therefore, we studied whether the secretion of cytokines changed according to the composition and structure of the material. After 48 h of culture, NO, pro-inflammatory cytokines (IL-2, IL-6, IL-17A, TNF-α and IFN-γ) and anti-inflammatory cytokines (IL-10) were detected by enzyme-linked immunosorbent assay. Both D-M1 and native D-CB significantly induced NO, IL-2, IL-6 and TNF-α production. D-DCB inhibited the secretion of these three cytokines and NO significantly, similar to M0, D-MC1 and D-MC2. In contrast, no scaffolds could induce change in the production of IL-10, IL-17A and IFN-γ significantly (Fig. 5). Taken together, while the expression of IL-10, IL-17A and IFN-γ is not changed, the expression of NO, IL-2, IL-6 and TNF-α of PM growth in D-CB is significantly inhibited after decellularization.

The results were similar to direct contact experiment, the expression of NO, IL-2, IL-6 and TNF-α of PM growth in I-CB is also significantly inhibited after decellularization. However, the expression of IFN-γ, IL-10 and IL-17A is changed but not significantly (Fig. 5).

### Proliferation of MSCs on scaffolds

**Figure 6:**
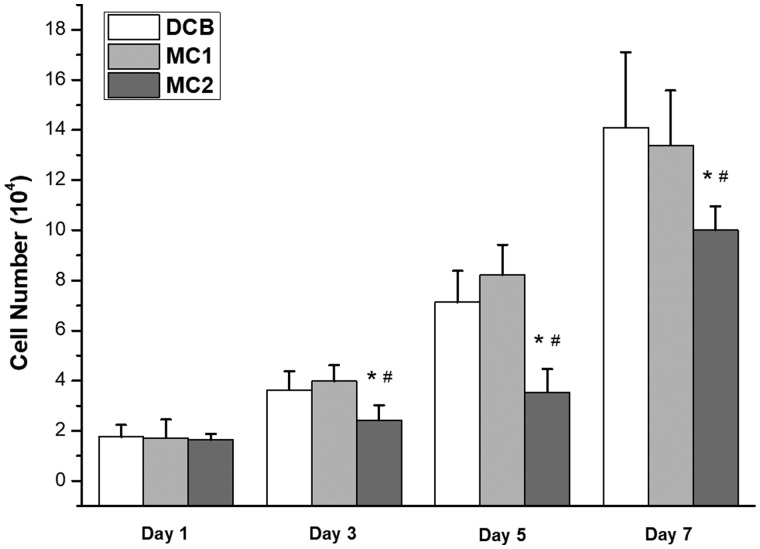
Number of MSCs cultured on scaffolds after 1, 3, 5 and 7 days, which show the proliferation of MSCs on scaffolds. (*P < 0.05 vs DCB#*P* < 0.05 vs MC1). cells seeded on CB were not detected, the number of cells significantly larger on the DCB and MC1 than that on MC2.

**Figure 7:**
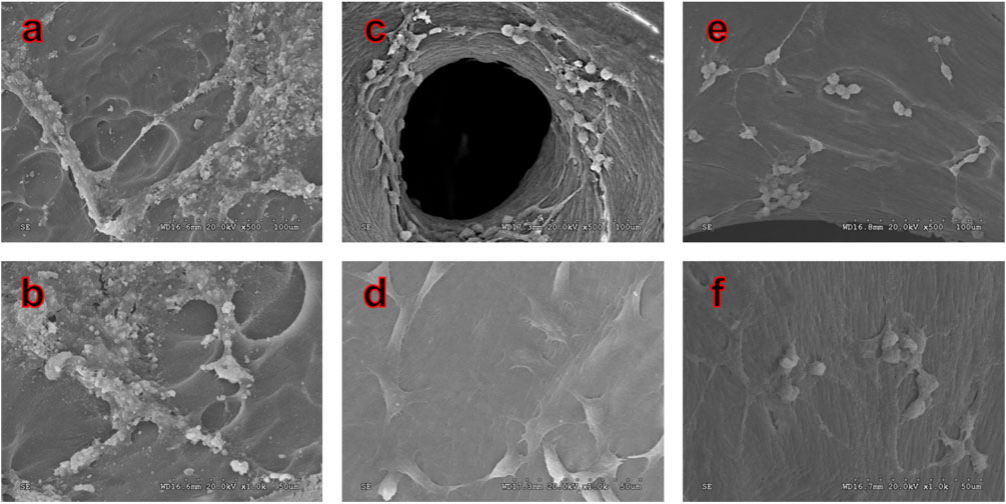
SEM Images show the MSCs cultured on fresh CB, DCB and MC1 after 7 days. (**a**, **b)** Fresh CB, (**c**, **d)** DCB, and (**e**, **f)** MC1, ×500 and ×1000 magnification. No cell adhesion was found in CB group. Cells grew well in DCB and MC1 group.

The number of cells on each scaffold was measured (Fig. 6). After 7 days of culture, the number of cells significantly larger on the DCB [(14.1 ± 3.0) × 10^4^/scaffold] and MC1 [(13.4 ± 2.2) × 10^4^/scaffold] than that on MC2 [(10.1 ± 1.0) × 10^4^/scaffold]. The number of cells on DCB is equal to that on MC1. However, cells seeded on CB were not detected during the experiment (data no show). On seventh day, no cell adhesion was found in CB group. In DCB group, more cells grew along the bone pores, there were intercellular connections and had grown in pieces, which indicated that the cells grew very well, and consistent with the results of cell counting. In MC1 group, more cells grew on the surface, which was similar to the DCB group’s (Fig. 7).

## Discussion

In order to improve the biocompatibility of nature bovine bone, a combined physicochemical decellularization method including EDTA, SDS and SC-CO_2_ treatments was applied in this study. After decellularization, the *in vitro* cytotoxicity and immunoreaction of bovine bone were very similar to the human decellularized bone product and bioceramic bone repair product. The chemical component and *in vitro* proliferation trend of osteogenic key cells on decellularized bovine bone was also similar to that of human decellularized bone products. These results indicated that the decellularized bovine bone in this study may have the potential to repair bone defect in clinic.

In decellularization process, it is difficult to keep the balance of between the complete removal of cellular residues and the retention of ECM component. And the decellularized bone must be biocompatible. EDTA can dissociate cells by subtly interfering with protein–protein interactions, but is insufficient for removing superficial cells even with agitation [35]. SDS can solubilize cell membranes from proteins, and remove cellular material [36]. Compared with other detergents (like Triton X-100), SDS is more effective to remove cell residues typically but with more disruptive effects on ECM [37]. Therefore, in this study, the SDS treatment process was only 6 h. In order to remove cell residues completely, a mild physical treatment was subsequently applied. Supercritical fluids have ideal transport properties, including high diffusivity, minimum surface tension and low viscosity as gases, but they also have the density and solvation ability similar to liquids, which are much higher than the density and solvation ability of most gases [38]. This combination of properties allows supercritical fluids like SC-CO_2_ to penetrate through surfaces easily without damaging them. In this study, 8 MPa was chosen as the minimum pressure above critical pressure (7.36 MPa). In order to remove the immunogenic substances effectively, scaffolds were exposed to a pressure of 15 MPa at 37°C for 10 min. About 37°C or approximate temperature has also been used for the study of acellular in other tissues, which can effectively remove immunogenic substances without causing damage to tissues [14, 39]. At the end of the procedure, DCB were washed in PBS extensively because it may be toxic to host cells if the chemicals used in the decellularization process remain.

After the combination of EDTA, SDS and SC-CO_2_ treatment, a large number of cellular remnants in fresh CB were effectively removed after decellularization, and thus the porosity of bovine bone was improved. Only a small amount of fiber can be found in the micropores, which may be fiber-like or residual bone marrow fibrous tissue (Fig. 2). However, the fiber-like materials in DCB did not have an obvious adverse effect on cellular compatibility (Table 2). The results of FTIR, Raman and XRD show that DCB is similar to commercial decellularized human bone, retains the effective components of collage, calcium and phosphate and provides biomechanical properties needed for body support and exercise (Figs 3 and 4). Cell surface oligosaccharide molecule α-Gal (Gal α 1,3-Galβ1-4GlcNAc-R) is a common antigen that can induce inflammation to biological scaffolds [18], which had been removed almost 100% in this study (Table 1). Treatment of nature bone to remove cellular remnants including Gal epitopes has been shown to minimize the adverse immune reactions *in vitro* (Fig. 5), which is consistent with others [40, 41]. The inflammatory response to implants is mainly mediated by macrophages. The ability of macrophages to express different functional phenotypes is usually shown under pathological conditions [42]. NO was compounded when macrophages were activated, which plays an important role in killing bacteria, microorganisms, tumor cells and organic foreign objects. Our data demonstrated that DCB could inhibit M1 polarization of macrophages *in vitro* compare with CB, which expresses lower levels of pro-inflammatory factors IL-2, IL-6 and TNF-α, and NO. The expression level of IL-17A and IFN-γ were also inhibited, but the expression level of anti-inflammatory IL-10 did not change significantly. The more inhibited expression of NO and pro-inflammatory factors, the weaker the cells’ immune response to the material. After decellularization, the immunity compatibility of bovine bone was significantly improved. However, the underlying mechanism by which DCB reduces the M1 macrophage phenotype is yet to be investigated. In order to evaluate the cytotoxicity of DCB comprehensively, multiple *in vitro* cytotoxicity tests including direct and indirect scaffolds-cells culture methods were applied. According to the cytotoxicity evaluation criteria of the United States Pharmacopoeia, the cytotoxicity results showed that the residual cytotoxic reagents were removed successfully after acellular treatment [43], and the cytotoxicity of DCB is equivalent to the commercial decellularized human bone and biological ceramic artificial bone (Table 2).

After confirming that DCB has a certain degree of biocompatibility, the proliferation property of MSCs implanted on scaffolds was evaluated *in vitro* (Figs 6 and 7). Because of the high cytotoxicity of CB, the planted MSCs could not survive. The proliferation of two kinds of decellularized natural bone (DCB and MC1) implanted MSCs is significantly better than that of bioceramic materials (MC2). Considering that DCB, MC1 and MC2 have similar cellular immune responses *in vitro*, it may be related to the unique microstructure and components of ECM that could support the proliferation of MSCs better. Our further work will focus on the effect of the microstructure of DCB on their cytocompatibility and its regulatory mechanism. These studies will help us to choose equally effective but simpler process, and prepare DCB for *in vivo* regenerative over the long term.

## Conclusion

In this study, a straightforward and combined decellularization process to generate xenogenous bone scaffolds with elimination of almost 100% a-Gal, retention of ECM microstructures and components, which significantly improved the biocompatibility. DCB is similar to commercial decellular human bone and can support the proliferation of MSCs. These results indicate the feasibility of decellularizing bone tissue with different species by using an appropriate combination of decellularization methodologies including SC-CO_2_ treatment. Our study has shown the potential application of this technique to generate a native decellularized scaffold for the clinical bone repair.

## Funding

This work was supported by National Natural Science Foundation of China (51502094), Special Fund Project for Guangdong Academy of Sciences to Build First-Class Research Institutions in China (2020GDASYL-20200103038), National Key R & D Plans (2018YFC1105902), China Postdoctoral Science Foundation (2017M612657), Guangdong Province Science and Technology Projects (2013B021800137), Pearl River Nova Program of Guangzhou (201610010168) and Guangdong Province Medical Research Foundation (A2016060).

## Ethical approval

This article does not contain any studies with human participants performed by any of the authors. All experimental protocols were approved by Ethics Committee on Experimental Animals of South China University of Technology. The animal experiments were complied with the ARRIVE guidelines and were carried out in accordance with the U.K. Animals (Scientific Procedures) Act, 1986 and associated guidelines.

*Conflict of interest statement*. The authors declared that they have no conflicts of interest to this work. We declare that we do not have any commercial or associative interest that represents a conflict of interest in connection with the work submitted. 
